# Liraglutide Attenuates Non-Alcoholic Fatty Liver Disease in Mice by Regulating the Local Renin-Angiotensin System

**DOI:** 10.3389/fphar.2020.00432

**Published:** 2020-04-08

**Authors:** Mengying Yang, Xiaoyi Ma, Xiuping Xuan, Hongjun Deng, Qi Chen, Li Yuan

**Affiliations:** Department of Endocrinology, Union Hospital, Tongji Medical College, Huazhong University of Science and Technology, Wuhan, China

**Keywords:** liraglutide, non-alcoholic fatty liver disease, the renin-angiotensin system, gluconeogenesis, inflammation, PI3K/AKT pathway

## Abstract

The renin-angiotensin system (RAS) is involved in the pathogenesis of non-alcoholic fatty liver disease (NAFLD) and represents a potential therapeutic target for NAFLD. Glucagon-like peptide-1 (GLP-1) signaling has been shown to regulate the RAS within various local tissues. In this study, we aimed to investigate the functional relationship between GLP-1 and the local RAS in the liver during NAFLD. Wild-type and ACE2 knockout mice were used to establish a high-fat-induced NAFLD model. After the mice were treated with liraglutide (a GLP-1 analogue) for 4 weeks, the key RAS component genes were up-regulated in the liver of NAFLD mice. Liraglutide treatment regulated the RAS balance, preventing a reduction in fatty acid oxidation gene expression and increasing gluconeogenesis and the expression of inflammation-related genes caused by NAFLD, which were impaired in ACE2 knockout mice. Liraglutide-treated HepG2 cells exhibited activation of the ACE2/Ang1-7/Mas axis, increased fatty acid oxidation gene expression, and decreased inflammation, which could be reversed by A779 and AngII. These results indicate that the local RAS in the liver becomes overactivated in response to NAFLD. Moreover, ACE2 knockout increases the severity of liver steatosis. Liraglutide has a negative and antagonistic effect on the ACE/AngII/AT1R axis, a positive impact on the ACE2/Ang1-7/Mas axis, and is mediated through the PI3K/AKT pathway. This may represent a potential new mechanism by which liraglutide improves NAFLD.

## Introduction

The renin-angiotensin system (RAS) is critical in the regulation of glycolipid metabolism and insulin sensitivity, which are closely related to metabolic syndromes [e.g., obesity, type 2 diabetes mellitus (T2DM), and non-alcoholic fatty liver disease (NAFLD)] (Marcus et al., 2013). Moreover, it has been reported that RAS inhibitors, as classical antihypertensive drugs, can effectively reduce the incidence of new-onset T2DM (Hermida et al., 2016). The RAS includes a classical angiotensin-converting enzyme (ACE)/angiotensin II (AngII)/type 1 angiotensin receptor (AT1R) axis and a new angiotensin-converting enzyme 2 (ACE2)/angiotensin 1-7(Ang1-7)/Mas axis, which has recently been found to antagonize with the classical axis (Mirabito et al., 2019). As a newly identified homologous ACE enzyme, ACE2 degrades AngII to produce Ang1-7 to coordinate the levels of AngII and Ang1-7. Functioning as two main biologically active peptides, AngII exerts pro-inflammation, pro-oxidation, pro-fibrosis, and vascular effects by activating AT1R, whereas Ang1-7 antagonizes most of the deleterious effects of AngII primarily *via* its Mas receptors.

The presence of a biologically active RAS in the liver is supported by the fact that multiple key components of the RAS have been identified in the liver (Herath et al., 2007; Zhang W. et al., 2013; Wu et al., 2016). Importantly, the two RAS axes identified both in circulation and locally in the liver are dramatically up-regulated in animal models of chronic liver diseases and in patients with chronic hepatitis and hepatic fibrosis (Lubel et al., 2009; Zhang W. et al., 2013). This indicates that liver injury induces excessive RAS activation; however, the precise role of the hepatic RAS in NAFLD remains poorly understood. More importantly, inhibition of the ACE/AngII/AT1R axis by AT1R inhibitors (Rosselli et al., 2009; Montez et al., 2012; Sansoe et al., 2019), gene knockout animal models (Jayasooriya et al., 2008), or activation of the ACE2/Ang1-7/Mas axis by exogenous Ang1-7 (Munoz et al., 2012; Feltenberger et al., 2013; Cao et al., 2016) in rodents can ameliorate NAFLD by suppressing liver lipogenesis, enhancing fatty acid oxidation, and inhibiting inflammation and gluconeogenesis. Taken together, the above studies demonstrate the essential participation of the RAS in NAFLD, indicating that inhibiting the ACE/AngII/AT1R axis or activating the ACE2/Ang1-7/Mas axis may represent effective targets for NAFLD treatment. However, the factors regulating the expression and activity of the RAS are largely unknown, and large research gaps remain.

There is emerging evidence supporting an association between glucagon-like peptide-1/glucagon-like peptide-1 receptor (GLP-1/GLP-1R) signaling and the RAS both in circulation and multiple local tissues (e.g., cardiovascular, lung, and kidney) (Zhang et al., 2015; Fandiño et al., 2018). As one of the most important incretins in the body, GLP-1 can exert hypoglycemic effects through various mechanisms, including promoting insulin secretion and islet beta cell proliferation, inhibiting apoptosis and glucagon secretion, suppressing appetite, and promoting satiety (Bifari et al., 2018). In addition, GLP-1R is widely distributed in a variety of tissues and organs (e.g., islet alpha and beta cells, cardiovascular system, kidney, and gastrointestinal tract) (Muller et al., 2019). GLP-1/GLP-1R signaling regulates the RAS through various signaling mechanisms, which play an essential role in the regulation of target tissue functions. It has been reported that the injection of exogenous GLP-1 in healthy individuals reduces circulating levels of AngII (Skov et al., 2013). In addition, liraglutide coordinates ACE and ACE2 expression in T1DM rat lung tissue, thereby down-regulating AngII and up-regulating Ang1-7 levels, protecting against AngII-mediated lung injury (Romaní-Pérez et al., 2015).

It has been well-established that both GLP-1 and the RAS are involved in the pathogenesis and treatment of NAFLD; however, whether GLP-1 ameliorates NAFLD *via* its interaction with the RAS and the functional correlation between GLP-1 and the RAS during NAFLD remains unknown. This study aimed to elucidate the physiological role of the RAS in GLP-1-mediated improvement of NAFLD, providing evidence for the treatment of NAFLD using GLP-1-based drugs.

## Materials and Methods

### Reagents and Antibodies

Liraglutide was obtained from Bachem (Torrance, CA, USA) and Exendin9-39 (Ex9-39, a GLP-1R antagonist) was purchased from California Peptide Research (Napa, CA, USA). Ang1-7, A779, and LY294002 (a PI3K inhibitor) were obtained from MedChem Express (MCE, New Jersey, USA). A triglyceride (TG) assay kit was obtained from BioRad (Richmond, CA, USA). Antibodies against β-actin, AKT, p-AKT (Ser473), PI3K, p-PI3K, AMPK, p-AMPK (Thr172), and NF-κB p65 were purchased from Cell Signaling Technologies (CST, Danvers, MA, USA). Antibodies against ACE were purchased from Abcam (Cambridge, UK) and anti-ACE2 was obtained from R&D Systems (Minneapolis, MN, USA). Other reagents were obtained from Sigma (St. Louis, MO, USA). Ang II, Ang1-7, and A779 were prepared in distilled PBS. LY294002 was dissolved in DMSO at 10 mmol/L stock solution. The final concentration of the vehicles did not exceed 0.1%.

### Treatment of Animals

Six-week-old male ACE2-knockout (ACE2KO) mice (C57BL/6J background) and their male wild type (WT) littermates were obtained from the Institute of Laboratory Animal Science, Chinese Academy of Medical Sciences and randomly divided into three groups (n = 7 per group): (1) standard diet control group (SD); (2) high-fat-diet induced NAFLD model group (HFD); and (3) liraglutide group (Lira). Mice in the SD group were fed a standard chow diet. The remaining mice were fed a HFD (60% of calories from fat; Research Diets, #D12492) for 3 months to induce NAFLD. Next, liraglutide (200 μg/kg/day) or an equivalent volume of sterile saline was intraperitoneally (i.p.) administered to the 19-week-old NAFLD mice for four weeks (Rahman et al., 2016; Luo et al., 2019).

The animals were housed under controlled light conditions (12 h light-dark cycle) with free access to food and water. Mouse experiments were performed according to procedures approved by the Animal Research Committee of Tongji Medical College, Huazhong University of Science and Technology, Wuhan, China. Body weight gain and fasting plasma glucose were monitored before and after the experiments.

### Glucose and Insulin Tolerance Tests

Mice were fasted overnight and a glucose tolerance test (GTT) was performed after an injection of glucose (2 g/kg body weight, i.p.). Blood glucose concentrations were measured using a OneTouch glucometer (LifeScan, Canada) both before (0 min) and after (30, 60, and 120 min) the glucose injections. For the insulin tolerance test (ITT), mice were fasted for 12 h and injected with recombinant human insulin (Roche, 0.75 U/kg body weight, i.p.). Glucose levels were measured both before (0 min) and after (15, 30, 60, and 90 min) the insulin injections. The area under the curve (AUC) was calculated by applying the trapezoidal rule (Xuan et al., 2018).

Plasma Ang1-7 concentration was evaluated using an Ang1-7 ELISA kit (Bio-Swamp, MU30979, China).

### Liver Dynamic ^18^FDG-PET/CT

Prior to PET imaging, the mice were fasted for 12 h. The mice were anesthetized with 2% isoflurane and placed on a scanning bed and approximately 200 ± 10 μCi 18-fluoro-6-deoxy-glucose (FDG) was intravenously injected. The PET/CT images were obtained immediately in dynamic mode for 60 min followed by a CT scan in normal mode using the TransPET Discoverist 180 system (Raycan Technology Co., Ltd, Suzhou, China). The PET images were reconstructed using the three-dimensional (3D) OSEM method with a voxel size of 0.5 × 0.5 × 0.5 mm^3^ (time frames: 10 s × 6, 30 s × 6, 1 min × 6, 5 min × 6, 10 min × 2). CT images were reconstructed using the FDK algorithm with the 256 × 256 × 256 matrix. Images were displayed using Carimas software (Turku PET Center, Turku, Finland). The mean standardized uptake value (SUV) was calculated as described previously (Liang et al., 2019), using the following formula: mean pixel value with the decay-corrected region-of interest activity (μCi/kg)/[injected dose (μCi)/weight (kg)].

### Histological Analysis and Triglyceride (TG) Content Assay

At the end of the experiments, all mice were sacrificed and the livers were weighed and stained with hematoxylin and eosin (H&E) and Oil Red O to evaluate the degree of hepatic steatosis and lipid deposition. Liver lipids were extracted from frozen liver tissues and the TG content from both the serum and liver tissues were measured using an enzyme-based assay kit (Sigma, St. Louis, MO). The TG values were normalized to the protein concentration.

### Cell Treatment

The HepG2 cell line is a human-derived hepatocellular carcinoma cell line, which is widely used to study the pathogenesis and clinical application of liver disease due to its similar biological activity with hepatocytes. Palmitic acid (PA), as a long-chain saturated fatty acid, is an important component of blood lipids, and plays an important role in the pathogenesis of lipid metabolism-related diseases, such as NAFLD. The HepG2 cell line was purchased from ATCC (American Type Culture Collection, Manassas, VA). HepG2 cells were cultured in DMEM containing 10% FBS in a 5% CO_2_ incubator at 37°C. The cell experiments were divided into three parts: (1) HepG2 cells were treated with or without PA (0.25 mmol/L) in the presence or absence of liraglutide (100 nmol/L) or Ex9-39 (10 μmol/L) for 24 h (Noyan-Ashraf et al., 2009; Li et al., 2019); (2) HepG2 cells were treated with PA (0.25 mmol/L) and/or liraglutide (100 nmol/L), A779 (10^-7^ mol/L), and AngII (100 nmol/L) for 24 h (Lu et al., 2014); and (3) HepG2 cells were treated with PA (0.25 mmol/L) and/or liraglutide (100 nmol/L) for 24 h with or without a pre-incubation with the PI3K inhibitor LY294002 (20 μmol/L) for 0.5 h (Liu et al., 2018).

### Real-Time Quantitative PCR (qPCR)

Total RNA was isolated from frozen hepatic tissue samples and cultured HepG2 cells using TRIzol reagent (Qiagen, Valencia, CA). The cDNA was synthesized using a PrimeScript RT reagent kit (Takara Biotechnology Co. Ltd., Japan) according to the manufacturer’s instructions. qPCR was performed in a LightCycler480-PCR machine (Roche Diagnostics, Mannheim, Germany). Objective gene expression was normalized to 18 s and analyzed by the 2^-ΔΔct^ method. The primer sequences are listed in **Table 1**.

**Table 1:** List of primers used for real-time PCR using SYBR-Green.

Gene	Forward sequence (5′→3′)	Reverse sequence (5′→3′)
ACE (m)	CTGAACCCCATTCCCAGTCC	AATTGACGCGGTTGGACTCT
ACE2 (m)	ACACTCTGGGAATGAGGACAC	ACACTCTGGGAATGAGGACAC
AT1R (m)	ATGGCTGGCATTTTGTCTGG	GTTGAGTTGGTCTCAGACAC
Mas (m)	GGCAGGATCTATTCCCAGAAGAA	CAGTCACCCTGGAACCAAGC
CPT-1a (m)	ACTCCGCTCGCTCATTCCG	CACACCCACCACCACGATAA
ACOX-1 (m)	AGGTTGTCATCGCTTTGG	GTGATTAACTCTGGATTGAAG
G6P (m)	AGACTCCCAGGACTGGTTCA	GTCCAGGACCCACCAATACG
PEPCK (m)	ACACCAATGGGGGTTTTGGT	AAAGGTAAGGAAGGGCGGTG
IκBα (m)	CCTGACCTGGTTTCGCTCTT	AGGTAAGCTGGTAGGGGGAG
NF-κB p65 (m)	CACCAAGGATCCACCTCACC	CTCTATAGGAACTATGGATACTGCG
IL-1β (m)	TGCCACCTTTTGACAGTGATG	AAGGTCCACGGGAAAGACAC
ACE (h)	CTCTTATTGGCCAGGGGACG	AAGTCCTGCAGTAGCCCAAC
AT1R (h)	GCGCGGGTTTGATATTTGACA	TCAAATACACCTGGTGCCGA
ACE2 (h)	CATTGGAGCAAGTGTTTGGATCTT	GAGCTAATGCATGCCATTCTCA
Mas (h)	GGGAATGCACATCGGCAAAT	CTCATCCGGAAGCACAGGAA
CPT-1a (h)	TTTGGACCGGTTGCTGATGA	TTTGGACCGGTTGCTGATGA
ACOX-1 (h)	GCTGGAGCTGCGGATTTAGA	AGCTTTTCTCGGGAAAGGAGGC
G6P (h)	CACTTCCGTGCCCCTGATAA	GTAGTATACACCTGCTGTGCCC
PEPCK (h)	AGTGATGGTGGCGTGTACTG	CTGGGACTGGAAACTGCAAAC
IκBα (h)	TGTGCTTCGAGTGACTGACC	TCACCCCACATCACTGAACG
NF-κB p65 (h)	TGGCCCCTATGTGGAGATCA	AGGGGTTGTTGTTGGTCTGG
IL-1β (h)	TGAGCTCGCCAGTGAAATGAT	TCCATGGCCACAACAACTGA
18s	GGA GAA CTC ACG GAC GAC GA	CCA GTG GTC TTG GTG TGC TG

ACE2, angiotensin-converting enzyme 2; CPT-1a, carnitine palmitoyltransferase 1a; ACOX1, acyl-CoA oxidase 1; G6P, glucose-6-phosphate isomerase; PEPCK, Phosphoenol pyruvate carboxykinase; NF-κB nuclear factor κB; (m), mouse; (h), human.

### Western Blotting

Protein from fresh-frozen liver tissues and cultured HepG2 cells were extracted in lysis buffer supplemented with phosphatase inhibitors (PhosSTOP, Roche, Germany). The supernatants were collected and the protein concentration was determined using a BCA assay (Life Technologies, USA). A western blot analysis of the tissue and cell culture extracts was performed according to standard procedures, and the membranes were incubated with primary antibodies against ACE, ACE2, AKT, p-AKT (Ser473), PI3K, p-PI3K, AMPK, and p-AMPK (Thr172) overnight at 4°C. Loading controls were established using β-actin immunoblot membrane staining. Blots were then washed three times with TBS-T and incubated with appropriate horseradish peroxidase-conjugated secondary antibodies (diluted 1:4,000 for all proteins) for 1 h in 5% skim milk at RT. The membranes were then washed three times in TBS-T for 5 min. Enhanced chemiluminescence (ECL) reagent (Millipore, USA) was used for signal detection.

### Immunofluorescence Staining

At the end of the treatment, HepG2 cells were fixed in 4% paraformaldehyde for 15 min and washed three times in PBS, and then incubated with a primary rabbit antibody against NF-κB p65 overnight at 4°C. After washing three times in PBS, the cells were incubated with an anti-rabbit FITC-conjugated secondary antibody. The nuclei were stained with DAPI and the cells were then observed under a microscope. Green fluorescence represents subcellular localization of NF-κB p65. The intensity of nuclear NF-κB staining in five different fields of each sample was analyzed using microcomputer-assisted Image J software.

### Statistical Analysis

All data are presented as the mean ± SD of at least three independent experiments. A one-way or two-way ANOVA with Bonferroni *post hoc* analysis was used for all statistical analyses. All analyses were performed using GraphPad Prism 7.0 (GraphPad Software, San Diego, CA, USA). P values < 0.05 were considered to be statistically significant.

## Results

### The Classical RAS Is Activated in NAFLD Mice

**Figure 1:**
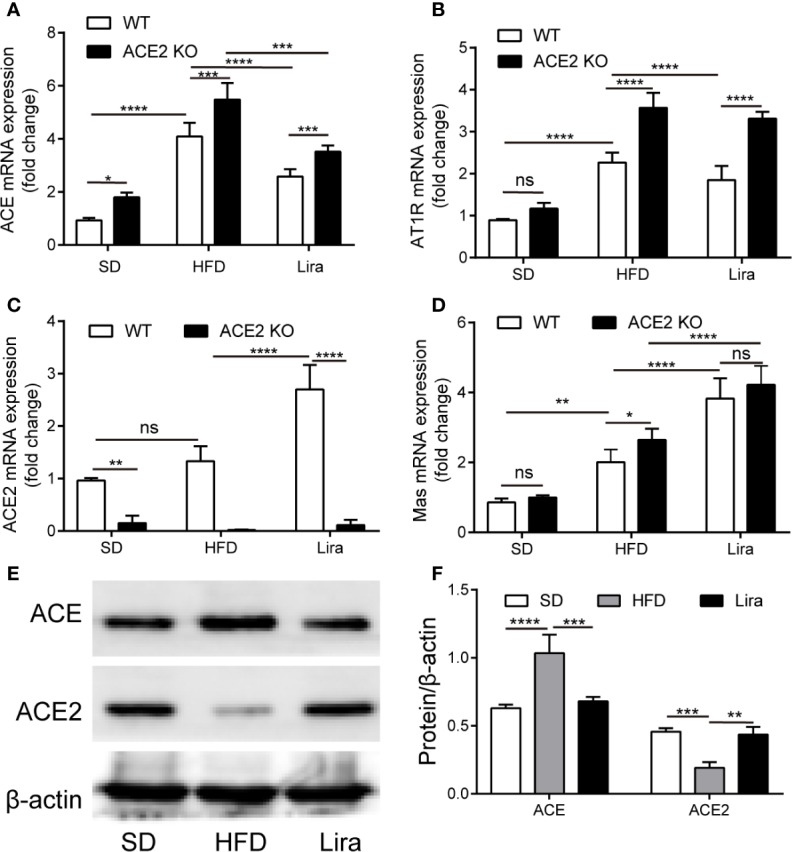
The regulation of liraglutide in the expression of the RAS in the liver. **(A–D)** Relative mRNA expression of ACE **(A)**, AT1R **(B)**, ACE2 **(C)**, and Mas **(D)** in livers of WT or ACE2KO mice treated with a HFD and/or liraglutide was measured by qPCR. **(E, F)** Representative western blotting images and analysis of ACE and ACE2 proteins in livers of WT mice treated with a HFD and/or liraglutide. n = 7. Data are expressed as the mean ± SD. Ns, no statistical difference; ^*^P < 0.05, ^**^P < 0.01, ^***^P < 0.001, and ^****^P < 0.0001. HFD, high fat diet; Lira, liraglutide; SD, standard diet; WT, wild type.

To evaluate whether the ACE/AngII/AT1R axis and ACE2/Ang1-7/Mas axis of the RAS were involved in NAFLD, we first examined its expression in the livers of NAFLD mice. We found that ACE and AT1R gene expression was markedly higher in the liver, and the ACE2 and its Mas receptor gene expression was also slightly higher in the mice that were fed a HFD for 16 weeks, compared to the SD-fed mice. (**Figures 1A–D**). Compared to the SD-fed mice, ACE protein expression was markedly higher in livers of the mice fed a HFD for 16 weeks, which was consistent with the corresponding gene expression, whereas the ACE2 protein content was decreased in NAFLD mice (**Figures 1E, F**). Moreover, in the ACE2KO mice, there was an accompanied deletion of the ACE2 gene and protein, and activation of the classical RAS signal was more pronounced in the HFD group (**Figures 1A, B**, **Supplementary Figures A, B**). Our results are consistent with previous studies in which the hepatic local RAS is up-regulated in animal models of liver injury (Sansoe et al., 2019).

### ACE2 Deficiency Aggravates Glucose Intolerance and Hepatic Steatosis in NAFLD Mouse Models

**Figure 2:**
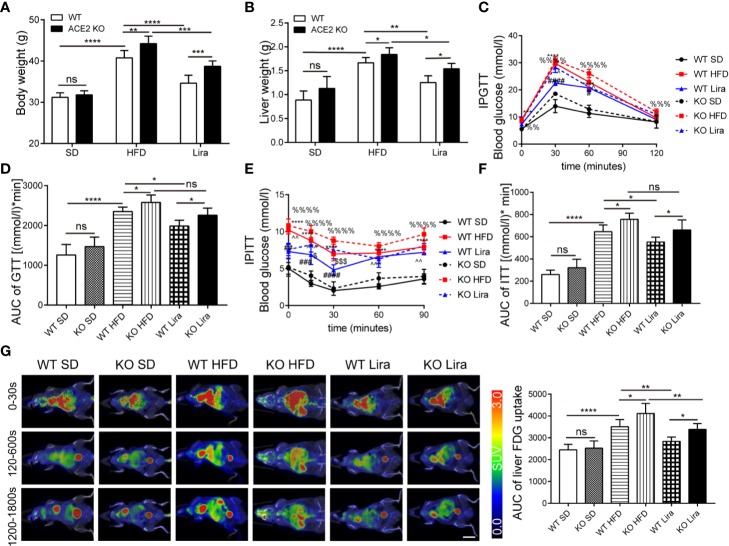
Liraglutide improves various metabolic parameters in NAFLD wild type (WT) mice, and the effect is weakened in ACE2KO mice. **(A)** Body weight. **(B)** Liver weight. **(C)** Glucose tolerance test (GTT) conducted in WT and ACE2KO mice and area under the curve (AUC) of the GTT **(D)**. **(E)** ITT conducted in WT and ACE2KO mice and AUC of the ITT **(F)**. n=7. **(G)** Representative 18-fluoro-6-deoxy-glucose (FDG)–PET images (left) and quantification of liver FDG uptake (right) for WT and ACE2KO mice after high fat diet (HFD) and/or liraglutide treatment. SUV, standardized uptake value. Scale bar, 10 mm. **(C, E)**: *P < 0.05 and **P < 0.01 vs. WT SD group; ^***^P < 0.001 and ^****^P < 0.0001 vs. WT SD group; ^#^P < 0.05, ^##^P < 0.01, ^###^ P < 0.001 and ^####^P < 0.0001 vs. WT HFD group; ^%%^P < 0.01 vs. KO SD group; ^%%%^P < 0.001 and ^%%%%^P < 0.0001 vs. KO SD group; ^^^P < 0.05 and ^^^^P < 0.01 vs. KO HFD group; ^^^P < 0.001 vs. KO HFD group and ^$^P < 0.05 and ^$$$^P < 0.001 vs. WT Lira group. NAFLD, non-alcoholic fatty liver disease; Ns, no statistical difference.

Given the changes in the RAS expression observed in NAFLD mice, we used ACE2 KO mice to study the role of the RAS in fatty liver formation, gluconeogenesis and inflammation. The mice fed a HFD showed a significant increase in body weight and liver weight compared to the control mice fed a SD, and this effect was further promoted by the ACE2 deficiency (**Figures 2A, B**). Consistent with our previous findings using GTT and ITT, the HFD mice exhibited impaired glucose tolerance and insulin resistance, the effect of which was more pronounced in ACE2KO mice (**Figures 2C–F**) (Xuan et al., 2018). Using ^18^FDG-PET/CT, we observed that the ACE2 depletion was associated with an increase in glucose uptake and metabolic activity in the liver, with a significant delay in hepatic glucose metabolism (**Figure 2G**).

**Figure 3:**
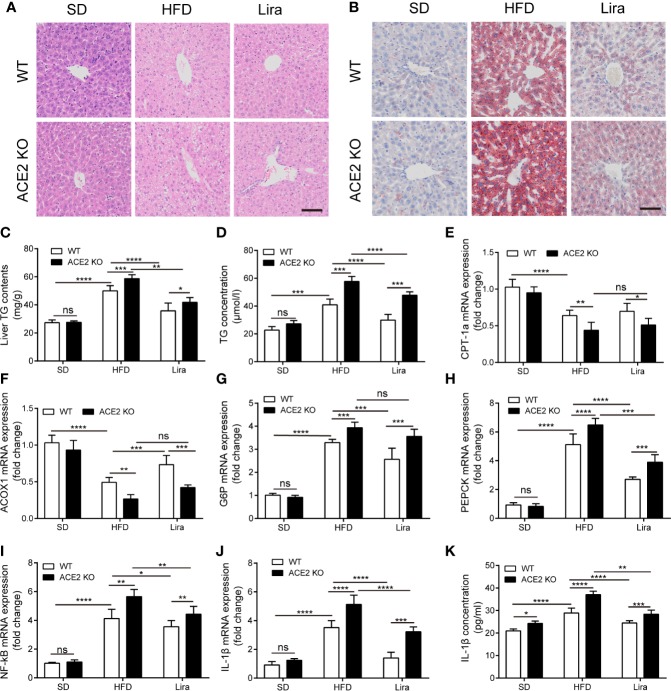
Liraglutide ameliorates hepatic steatosis with suppressed gluconeogenesis and inflammation in non-alcoholic fatty liver disease (NAFLD) wild type (WT) mice. HE staining **(A)** and Oil red O staining **(B)** of liver tissues; Scale bars = 50 μm. **(C)** the statistical charts of TG content in liver tissues; **(D)** Serum TG concentration. **(E, F)** Relative mRNA expressions of fatty acid oxidation-related genes ACOX-1 **(E)** and CPT-1a **(F)** in livers were measured by quantitative PCR (qPCR). **(G–J)** Relative mRNA expressions of gluconeogenesis-related genes G6P **(G)** and PEPCK **(H)**, as well as inflammation-related genes NF-κB **(I)** and IL-1β **(J)** in livers. **(K)** Serum IL-1β concentration. n=7. Data are expressed as the mean ± SD. ^*^P < 0.05, ^**^P < 0.01, ^***^P < 0.001, and ^****^P < 0.0001. Ns, no statistical difference.

Hepatic steatosis was evaluated by both H&E and Oil red O staining. The livers in the NAFLD group exhibited profound lipid accumulation within the hepatocytes compared to the SD group, which was aggravated in ACE2KO mice (**Figures 3A, B**). These observations were further confirmed by a marked increase in the TG content in NAFLD livers and serum compared to the SD group, whereas there was markedly increased hepatic TG content in ACE2KO mice (**Figures 3C, D**). In HFD-induced NAFLD, liver lipolysis enzyme-related gene expression was decreased (**Figures 3E, F**), and gluconeogenesis and inflammatory gene expression were increased (**Figures 3G–J**). A loss of ACE2 expression in the liver significantly decreased the expression of genes involved in fatty acid degradation in the mouse livers (P < 0.05) (**Figures 3E, F**). The expression of genes associated with gluconeogenesis and inflammation in the liver of NAFLD ACE2 KO mice was also substantially increased (**Figures 3G–J**). The concentration of the inflammatory factor IL-1β in serum also increased in WT mice fed a HFD, and increased even more in ACE2 KO mice fed a HFD (**Figure 3K**). These results suggest that an ACE2 deficiency aggravates glucose intolerance and hepatic steatosis in NAFLD.

### Liraglutide Regulates RAS Component Expression in the Livers of NAFLD Mice

It has been reported that GLP-1 is closely linked to RAS in multiple organs (Zhang et al., 2015; Fandiño et al., 2018). Therefore, we investigated the liraglutide-mediated regulation of RAS system to identify the role of the RAS in NAFLD progression and improvement. As shown in **Figures 1A–D**, a qPCR analysis of the gene expression of RAS components revealed a marked down-regulation of ACE and AT1R, as well as an upregulation of ACE2 and Mas mRNA levels in the hepatic tissues of the liraglutide group compared with the NAFLD mice. In addition, liraglutide increased the expression of ACE2 protein, decreased the expression of ACE protein, and restored RAS balance in the liver (**Figure 1E**). In the mice fed with a HFD, we observed the concentration of Ang1-7 decreased compared to the SD group. While, the decreased Ang1-7 levels was increased by the treatment of liraglutide (**Supplementary Figure E**). On the whole, these results demonstrate that there is a close regulatory relationship between GLP-1 and the RAS in the liver.

### Liraglutide Ameliorates Hepatic Steatosis and Inflammation in NAFLD Mice

We further explored the treatment of liraglutide in obesity-induced insulin resistance and liver steatosis in the liver. We first analyzed the metabolic phenotypes of mice treated with a HFD and/or liraglutide in WT mice. The body and liver weight in the NAFLD group were all higher than those of the SD mice, whereas the administration of liraglutide prevented the increase in the parameters induced by NAFLD, and there were no statistical differences in body and liver weight between the liraglutide group and SD group (**Figures 2A, B**). Consistently, GTT and ITT in the HFD mice showed improved glucose control after four weeks of liraglutide treatment compared to the HFD mice treated with saline, which was similar to that of the SD group. (**Figures 2C–F**). By using ^18^FDG-PET/CT, we observed that the HFD substantially decreased glucose metabolism and decreased metabolic activity in the liver (**Figure 2G**). Liraglutide promoted glucose metabolism in the liver, and glucose in the liver was almost completely metabolized within 30 min; there were no statistical differences in glucose metabolism between the liraglutide group and the SD group (**Figure 2G**). In addition, as shown by H&E and Oil Red O staining, liver lipid accumulation, as well as the liver and serum TG levels were significantly ameliorated in the liraglutide group following liraglutide administration compared to the HFD group (**Figures 3A–D**).

### ACE2 Deletion Reduces the Protective Effect of Liraglutide in the Liver

Our findings confirmed a correlation between GLP-1 and the RAS in the liver, and that the GLP-1 analogue, liraglutide, improves fatty liver. However, it remained unknown whether liraglutide directly improves hepatic steatosis and inflammation through the local RAS in the liver. ACE2KO mice were injected with liraglutide or saline for four weeks. As shown in **Figure 2**, GLP-1 improved insulin resistance and fatty liver in WT mice fed a HFD; however, this effect was not significant in ACE2KO mice fed a HFD (**Figures 2C–F**). Liver H&E and Oil red O staining revealed that the livers of ACE2KO HFD mice had significant higher fatty deposits compared to the WT group fed a HFD, even after the same liraglutide treatment (**Figures 3A, B**). Fatty acid β oxidation gene (ACOX-1 and CPT-1a) expression was lower in liraglutide-treated ACE2KO mouse livers compared to liraglutide-treated WT mice livers (**Figures 3E, F**), whereas the expression of gluconeogenesis-related genes (G6P and PEPCK) and inflammatory genes (IL-1β and NF-kB) exhibited the opposite effect (**Figures 3G–J**). The serum IL-1β concentration in liraglutide-treated ACE2KO mice was higher than in the liraglutide-treated WT mice (**Figure 3K**). These results indicate that the RAS plays an important role in liraglutide-mediated improvement of hepatic steatosis in NAFLD mice.

### Liraglutide Regulates RAS Component Expression in PA-Induced HepG2 Cells

**Figure 4:**
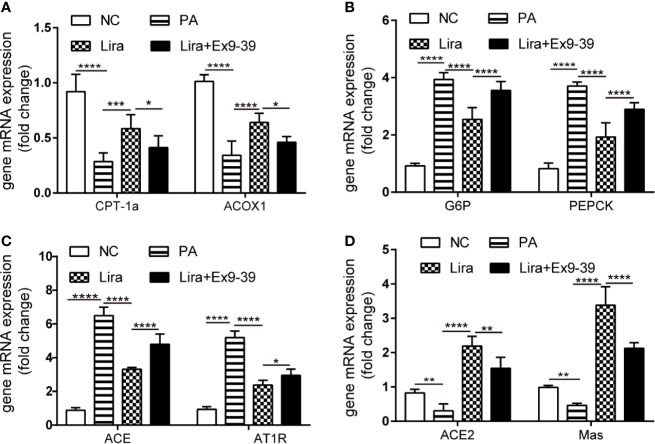
Liraglutide regulates the expression of the RAS in HepG2 cells, which is reversed by Ex9-39. HepG2 cells were treated with or without PA (0.25 mmol/L) in the presence or absence of liraglutide (100 nmol/L) or Ex9-39 (10 μmol/L) for 24 h. Relative mRNA expression of CPT-1a, ACOX1 **(A)** and gluconeogenesis genes (G6P and PEPCK) **(B)** as well as RAS components (ACE, AT1R, ACE2, and Mas gene) **(C, D)** were measured by qPCR in HepG2 cells. n=3. Data are expressed as the mean ± SD. ^*^P < 0.05, ^**^P < 0.01, ^***^P < 0.001, and ^****^P < 0.0001. NC, normal control group.

In *in vitro* experiments, HepG2 cells were induced with 0.25 mmol/L PA exposure for 24 h with or without liraglutide and Ex9-39, a specific inhibitor of GLP1R (**Figure 4**). PA decreased the expression of fatty acid β oxidation genes (ACOX-1 and CPT-1a) and increased the expression of G6P and PEPCK genes, which were reversed by liraglutide and rescued by Ex9-39. There were no statistical differences in mRNA levels between the liraglutide + Ex9-39 group and PA-treated group (**Figures 4A, B**). Interestingly, PA-induced HepG2 cells treated with liraglutide downregulated the gene expression of ACE and AT1R, while concomitantly upregulating ACE2 and Mas mRNA levels compared to the PA group (**Figures 4C, D**), which was consistent with the *in vivo* results (**Figure 1**). While, the mRNA level changes of ACE, AT1R, ACE2 and Mas regulated by liraglutide were rescued by Ex9-39 in the liraglutide + Ex9-39 group (**Figures 4C, D**). This suggests that liraglutide regulates both the ACE/Ang II/AT1R axis and the ACE2/Ang1-7/Mas axis under NAFLD conditions, both *in vivo* and *in vitro*.

### Blockage of the ACE2/Ang1-7/Mas Axis Attenuates the Liraglutide-Mediated Benefits of Hepatocellular Steatosis in HepG2 Cells

**Figure 5:**
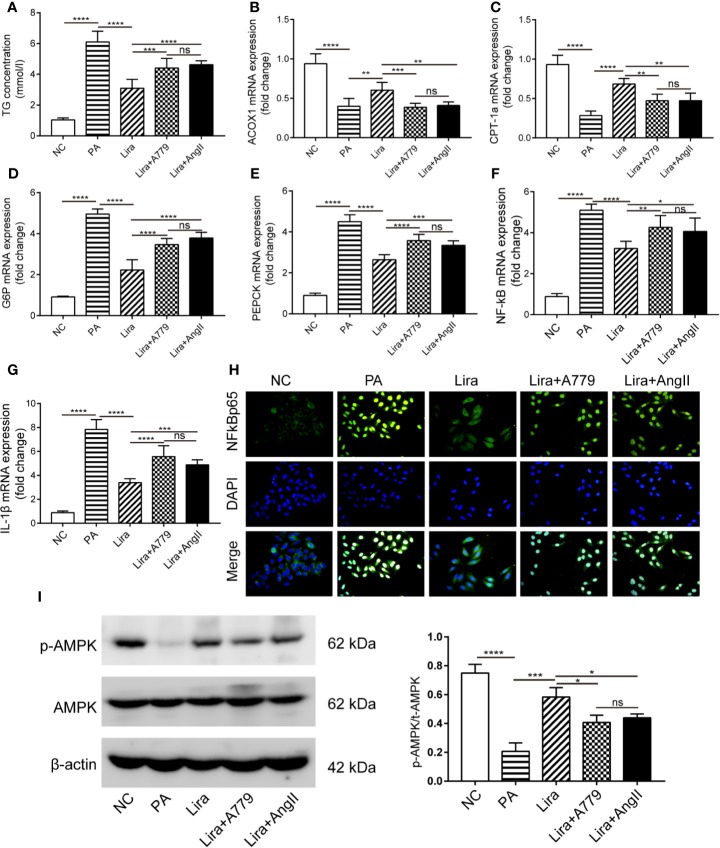
A blockage of the ACE2/Ang1-7/Mas axis attenuates liraglutide-mediated benefits on hepatocellular steatosis and gluconeogenesis, as well as inflammation and related AMPK signaling pathways in HepG2 cells. HepG2 cells were treated with or without PA (0.25 mmol/L) in the presence or absence of liraglutide (100 nmol/L) or liraglutide (100 nmol/L) + A779 (10^-7^ mol/L) combination or liraglutide (100 nmol/L) + AngII (100 nmol/L) for 24 h. **(A)** The statistical charts of TG content in HepG2 cells. **(B–G)** Relative mRNA expression of ACOX-1 **(B)**, CPT-1a **(C)** and G6P **(D)**, PEPCK **(E)** as well as NF-κB **(F)**, IL-1β **(G)** in HepG2 cells was measured by qPCR; **(H)** Nuclear translocation of NF-κB p65 in HepG2 cells was measured by immunofluorescence staining; magnification, 400. **(I)** Western blotting of AMPK phosphorylation in HepG2 cells. n=3. Data are expressed as the mean ± SD. ^*^P < 0.05, ^**^P < 0.01, ^***^P < 0.001, and ^****^P < 0.0001. Ns, no statistical difference.

The above results demonstrate the regulation of RAS component expression by liraglutide both *in vivo* and *in vitro*. To determine whether the liraglutide-mediated regulation of the ACE2/Ang1-7/Mas axis and ACE/AngII/AT1R axis, as well as its beneficial effects on HepG2 cells is of functional relevance, we explored the influence of pharmacological inhibition of the ACE2/Ang1-7/Mas axis or activation of the ACE/AngII/AT1R axis on the liraglutide-mediated benefits on hepatocellular steatosis using A779 (specific inhibitor of Mas) or AngII. Lipid accumulation in HepG2 cells was examined using a TG content assay. PA significantly promoted the intracellular TG content, and liraglutide inhibited PA-induced lipid accumulation (**Figure 5A**). Importantly, the liraglutide-mediated inhibition of TG content was largely abrogated by the ACE2/Ang1-7/Mas blockage through the addition of A779. Additionally, the TG content increased in liraglutide combined with the AngII group compared with the liraglutide individual intervention group. There were no statistical differences in TG content between the liraglutide+A779 group and liraglutide+AngII group (**Figure 5A**). We detected the expression of the ACOX-1 and CPT-1a genes. ACOX-1 and CPT-1a mRNA expression was downregulated in PA-treated cells compared with the negative control group, which was prevented by liraglutide treatment (**Figures 5B, C**). The addition of A779 or AngII largely blunted the promotion of liraglutide on ACOX-1 and CPT-1a expression; there were no statistical differences in the expression of these genes between the liraglutide+A779 group, the liraglutide+AngII group and the PA group (**Figures 5B, C**).

### Blockage of the ACE2/Ang1-7/Mas Axis Attenuates Liraglutide-Mediated Inhibition of Gluconeogenesis and Inflammation in HepG2 Cells

Similarly, we detected gluconeogenesis and inflammation-related gene expression. A significant increase of G6P and PEPCK mRNA levels following PA exposure was observed (**Figures 5D, E**). As expected, liraglutide treatment suppressed such increases (**Figures 5D, E**). Importantly, A779 and AngII strikingly alleviated the inhibition of liraglutide on G6P and PEPCK expression (**Figures 5D, E**). Regarding the NF-κB/IL-1β pathway, PA-mediated increases in NF-κB and IL-1β mRNA levels were largely prevented by liraglutide (**Figures 5F, G**). Additionally, the liraglutide-mediated prevention of NF-κB/IL-1β mRNA upregulation induced by PA was blunted in the presence of A779 or AngII (**Figures 5F, G**). The mRNA levels of G6P, PEPCK, NF-κB, and IL-1β of the lira + A779 group and lira +AngII group were lower those of the PA group, but the degree of decline was not significant compared to those of the liraglutide group (**Figures 5D–G**). The nuclear translocation of NF-κB p65 in HepG2 cells was determined by immunofluorescence. The results in **Figure 5H** demonstrate that more NF-κB p65 was localized in the nucleus of the PA group compared to that in the NC group. Moreover, liraglutide prevented the PA-induced nuclear translocation of NF-κB p65, whereas liraglutide failed to prevent this effect when co-treated with A779 or AngII (**Figure 5H**). These results suggest that the beneficial consequences of liraglutide on hepatocellular steatosis and gluconeogenesis, as well as inflammation, were at least partially dependent on liraglutide-mediated upregulation of the ACE2/Ang1-7/Mas axis and downregulation of the ACE/AngII/AT1R axis.

### Blockage of the ACE2/Ang1-7/Mas Axis Attenuates Liraglutide-Mediated Activation on AMPK Activity in HepG2 Cells

Having observed enhanced fatty acid β oxidation and inhibited gluconeogenesis in response to liraglutide and Ang1-7, possible mechanisms responsible for these beneficial effects were identified. AMPK is a metabolic regulator that is implicated in the regulation of glycolipid metabolism and inflammation in the liver. Activation of AMPK is reportedly associated with enhanced fatty acid oxidation and inhibition of gluconeogenesis and inflammation (Koo et al., 2005; Smith et al., 2016). Accordingly, AMPK activity was examined in HepG2 cells by western blot. The protein level of p-AMPKα was inhibited by PA intervention (**Figure 5I**). Treatment with liraglutide remarkedly increased the p-AMPKα level, and the addition of A779 or AngII almost blocked liraglutide-mediated upregulation of AMPKα phosphorylation (**Figure 5I**).

### Liraglutide Regulates RAS Activity Through the PI3K/AKT Pathway in PA-Induced HepG2 Cells

**Figure 6:**
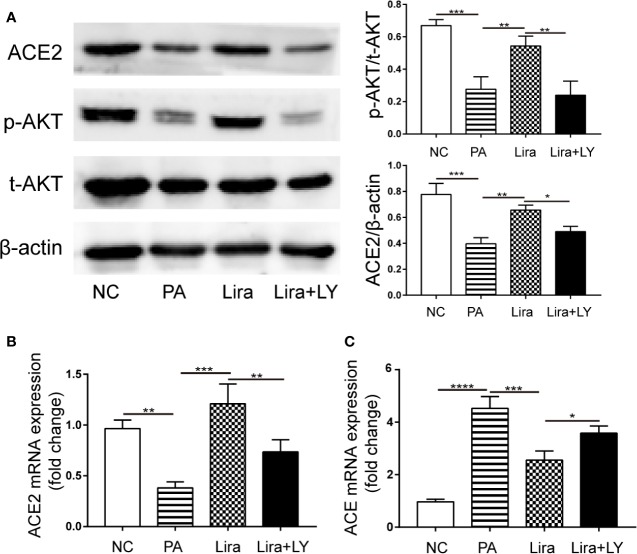
The regulation of liraglutide on the expression of the key renin-angiotensin system (RAS) components involved in the PI3K/AKT pathway in PA-induced HepG2 cells. HepG2 cells were pre-incubated for 0.5 h with PI3K inhibitor LY294002 (20 μmol/L), followed by co-incubation with liraglutide (100 nmol/L) and PA (0.25 mmol/L) for 24 h. **(A)** Representative western blotting images of AKT phosphorylation and ACE2 proteins in HepG2 cells (left) and statistical analysis (right). **(B, C)** Relative mRNA expression of ACE2 **(B)** and ACE **(C)** in HepG2 cells was measured by qPCR. n=3. Data are expressed as the mean ± SD. ^*^P < 0.05, ^**^P < 0.01, ^***^P < 0.001, and ^****^P < 0.0001.

To investigate the molecular mechanism of liraglutide activity on RAS component expression, the PI3K/AKT pathway was assessed, which has been implicated in various effects of GLP-1. In islet beta cells, GLP-1 promotes beta cell proliferation, inhibits apoptosis, and regulates beta cell differentiation by activating the downstream PI3K/AKT pathway (Shao et al., 2014). Accordingly, we hypothesized that the PI3K/AKT pathway was also involved in liraglutide-mediated regulation of RAS component expression in HepG2 cells. To test this hypothesis, HepG2 cells were preincubated with LY294002, a specific inhibitor of PI3K for 0.5 h. The total protein expression levels of PI3K and AKT, as well as phosphorylated PI3K and AKT in HepG2 were analyzed by Western blot, indicating that liraglutide could also activate the PI3K/AKT pathway in HepG2 cells (**Figure A**, **Supplementary Figures C, D**). Next, we examined the expression of ACE2 protein and RAS component genes. The results showed that the upregulation of ACE2 mRNA and protein by liraglutide were all inhibited by LY294002, with the opposite trend observed for the ACE gene, and the levels of p-AKT and ACE2 protein as well as ACE and ACE2 mRNA were similar between the LY294002 pretreatment group and PA-treated group (**Figures 6A–C**). This finding suggested that liraglutide-mediated regulation of the RAS in HepG2 cells is largely required for activation of the PI3K/AKT signaling pathway.

## Discussion

The GLP-1/RAS axis has been identified in the present study. Liraglutide as a GLP-1 analogue was found to ameliorate NAFLD, at least partially, by modulating the expression and activity of the hepatic RAS. The main findings of our study are as follows: (1) the RAS is important for glucose metabolism and liver function, as well as significant liver metabolic disorder in ACE2-deficient mice; (2) liraglutide ameliorated NAFLD in mice, which was associated with enhanced fatty acid β-oxidation, as well as suppression of gluconeogenesis and inflammation in the liver associated with NF-κB activation, which was attenuated in ACE2KO mice; (3) in both the *in vivo* and *in vitro* NAFLD models, the hepatic local RAS was over-activated. Treatment with liraglutide down-regulated ACE and AT1R while further up-regulating ACE2 and Mas expression. These findings indicate that liraglutide plays a dual regulatory role on the two axes of the RAS; (4) the RAS axis could be reversed following treatment with A779 or AngII, which attenuated or even blocked liraglutide-mediated improvement of NAFLD. These findings suggest that the ACE2/Ang1-7/Mas axis is essential for liraglutide-mediated amelioration of NAFLD, and that the PI3K/AKT pathway is the signaling mechanism through which liraglutide regulates the RAS.

Our results have shown that the expression of ACE and AT1R mRNA in the liver was dramatically up-regulated (**Figure 1**), indicating that the hepatic local RAS was over-activated during NAFLD. Numerous studies have considered that the expression and activity of the classical RAS in both the circulation and locally in the liver are up-regulated in response to liver injury (e.g., patients with chronic hepatitis and liver fibrosis (Kim et al., 2016), as well as animal models of liver disease (Montez et al., 2012; Kim et al., 2016; Sansoe et al., 2019)). In rat models of CCL4-induced hepatic fibrosis and fructose fed-NAFLD (Zhang W. et al., 2013; Frantz et al., 2017), the hepatic RAS was over-activated and imbalanced, and the ratios of ACE/ACE2, AngII/Ang1-7, and AT1R/Mas were upregulated. The upregulation of the ACE2/Ang1-7/Mas axis may function as a compensatory mechanism to oppose the deleterious actions of AngII over-production; however, the compensatory increase in Ang1-7 appears to be insufficient to antagonize the elevated AngII levels, ultimately leading to the progression of liver disease. However, little is currently known about the factors associated with RAS activation. Previous studies have found that for high-glucose upregulated angiotensinogen (AGT), ACE, and AT1R gene expression in rat proximal tubular cells (Zhou et al., 2010; Wang et al., 2017) and livers (Gabriely et al., 2001), insulin could inhibit high glucose-induced AGT upregulation by acting on insulin response elements on the AGT gene promoter; however, this effect was not observed in insulin-resistant rats. The findings of the above studies combined with our results suggest that with NAFLD, the combination of glucolipotoxicity and insulin resistance may activate the hepatic RAS, although the associated molecular mechanisms remain unknown.

The dual regulation of liraglutide on the ACE/AngII/AT1R and ACE2/Ang1-7/Mas axes was found to be partially mediated *via* PI3K/AKT signaling (**Figure 6**). Previous studies have established an association between GLP-1 signaling and the RAS in multiple organs, in which they modulate target organ functions through various pathways. In vascular mesangial cells (Ishibashi et al., 2012) and vascular smooth muscle cells (Zhao et al., 2014), GLP-1 antagonized AngII-mediated activation of NF-κB signaling and oxidative stress by activating the cAMP/PKA pathway, thereby preventing AngII-induced cell damage and senescence. The following two main downstream pathways of GLP-1R have been well-characterized in islet β cells: (1) activation of adenylate cyclase, leading to elevated cAMP and subsequent PKA activation; and (2) activation of the PI3K/Akt pathway (Shao et al., 2013; Shao et al., 2014). Our investigation extends the mechanisms underlying the association between GLP-1 and the RAS as supported by PI3K/AKT signaling being essential for liraglutide-mediated modulation of the hepatic RAS. Another recent study of GLP-1-mediated regulation of the RAS deserves attention. Pan et al. (Pan et al., 2018) reported that fibroblast growth factor 21 (FGF21) up-regulated ACE2/Ang1-7 expression and production in both adipocytes and the kidney, preventing AngII-induced vascular dysfunction. In another study (Lee et al., 2014), GLP-1R activation exerted benefits on NAFLD by up-regulating FGF21 and its receptor axis in the liver. These two studies combined with our results indicate that the up-regulation of the FGF21/FGF21R axis by GLP-1 may be one of the mechanisms by which liraglutide regulates the RAS in the liver.

The therapeutic effects of GLP-1-based drugs on NAFLD have been well-established in several recent studies (Ben-Shlomo et al., 2011; Mells et al., 2012). Moreover, GLP-1R expression has been identified in the hepatocytes of both rodents and humans (Gupta et al., 2010). GLP-1 also exerts therapeutic effects on NAFLD through various mechanisms, including ameliorating insulin resistance, inhibiting lipogenesis, and enhancing fatty acid β-oxidation (Ding et al., 2006; Aroor et al., 2015), as well as attenuating gluconeogenesis (Lee et al., 2007) and inflammation (Zhang L. et al., 2013). Corroborating these data, our findings confirmed the benefits of liraglutide on NAFLD. In particular, liraglutide improved fatty acid oxidation and inhibited both gluconeogenesis and NF-κB/IL-1β inflammation signaling (**Figure 2**), which were associated with AMPK activation (**Figure 5**).

In this study, we also observed that ACE2 knockout can aggravate HFD-induced liver fat deposition, as well as promote gluconeogenesis and NF-κB/IL-1β inflammatory signaling (**Figure 3**). These results are consistent with the conclusions obtained in obese rats treated with Ang1-7 (Santos et al., 2013) and transgenic rats overexpressing Ang1-7 (Bilman et al., 2012), which alleviate hepatic steatosis and TLR4/MAPK/NF-κB signaling activity inhibition. Such inhibition is associated with an improvement of glucose metabolism combined with inhibited gluconeogenesis in the liver. ACE2 deficiency leads to an over-activation of the classical RAS signaling axis and may be one of the causes of liver damage and aggravation of glucose intolerance (**Figures 1** and **2**). A previous study in transgenic rats with elevated plasma AngII levels (Wei et al., 2009) indicated that AngII-induced NAFLD is primarily caused by oxidative stress-mediated mitochondrial dysfunction and impaired mitochondria-mediated fatty acid β oxidation *via* AT1R. In another study (Ishizaka et al., 2011), AngII injection in rats inhibited AMPK activity and suppressed fatty acid oxidation gene (PPARα and CPT-1a) expression without altering liver lipogenesis-related gene expression. The results of this study further emphasize the importance of the ACE2/Ang1-7/Mas and ACE/AngII/AT1R axes balance in the treatment of NAFLD.

As two major metabolic signals regulating glycolipid metabolism, both GLP-1 and the RAS target islet and insulin within peripheral target organs (e.g., liver, skeletal muscle, and adipose tissue) through common or cross-reactive pathways (Leung and de Gasparo, 2009; Rodriguez et al., 2019). In addition, GLP-1-based drugs function as a new class of hypoglycemic drugs, and RAS inhibitors act as a classical class of anti-hypertensive drugs, which have also been confirmed to have anti-diabetic effects. What would be the therapeutic benefits of combining the two drugs? Studies in T2DM model mice (Miyagawa et al., 2013) have demonstrated that co-treatment with vildagliptin (a DPP4 inhibitor) and valsartan (an AT1R inhibitor) synergistically reduced blood glucose levels primarily through synergistically improving the insulin secretion of islets, as well as amelioration of fatty acid oxidation in the liver. In addition, Wang et al. (Wang et al., 2011) identified the IRS-2/PI3K/AKT/FOXO1 signaling pathway as the common downstream target of GLP-1 and the RAS, which was responsible for the protective effect of both drugs on islet beta cells. However, the synergistic effects and precise mechanism of GLP-1 and RAS-related drugs on NAFLD treatment remains unknown, and further research is needed in the future.

In conclusion, we identified that the RAS is over-activated during NAFLD and that the hepatic RAS is a critical intermediary for liraglutide in liver protection. Liraglutide down-regulates the ACE/Ang II/AT1R axis, exhibits positive effects on the ACE2/Ang1-7/Mas axis though the PI3K/AKT pathway, and antagonizes hepatocellular steatosis. These findings provide a novel insight into the mechanism of liraglutide in NAFLD therapy.

## Data Availability Statement

All datasets generated for this study are included in the article/**Supplementary Material**.

## Ethics Statement

The animal study was reviewed and approved by the Animal Research Committee of Tongji Medical College, Huazhong University of Science and Technology, Wuhan, China.

## Author Contributions

MY, XM, and HD were involved in designing and performing experiments *in vivo*. QC and MY performed the *in vitro* experiments. XM and MY were involved in the acquisition of data. XM and XX performed statistical analysis and interpretation of data. MY and XM wrote sections of the manuscript. LY contributed to critical revision of the manuscript and final approval for publication.

## Funding

This work was supported by the National Natural Science Foundation of China (grant numbers 81570700, 81974104).

## Conflict of Interest

The authors declare that the research was conducted in the absence of any commercial or financial relationships that could be construed as a potential conflict of interest.

## Supplementary Material

Supplementary figure **(A, B)** Representative ACE and ACE2 Western blotting images **(A)** and analysis of ACE protein in livers of ACE2KO mice treated with a HFD or/and liraglutide **(B)**. **(C, D)** Representative Western blotting images of PI3K phosphorylation protein in HepG2 cells **(C)** and statistical analysis **(D)**. **(E)** The plasma concentration of Ang1-7 of WT mice treated with a HFD or/and liraglutide. Data are expressed as the mean ± SD. Ns, no statistical difference; ^*^P < 0.05 and ^**^P < 0.01.
