# Supplementary figures and images for: Liraglutide Attenuates Non-Alcoholic Fatty Liver Disease in Mice by Regulating the Local Renin-Angiotensin System
Source: Front Pharmacol. 2020 Apr 8;11:432. doi: 10.3389/fphar.2020.00432 (PMC7156971; doi:10.3389/fphar.2020.00432)

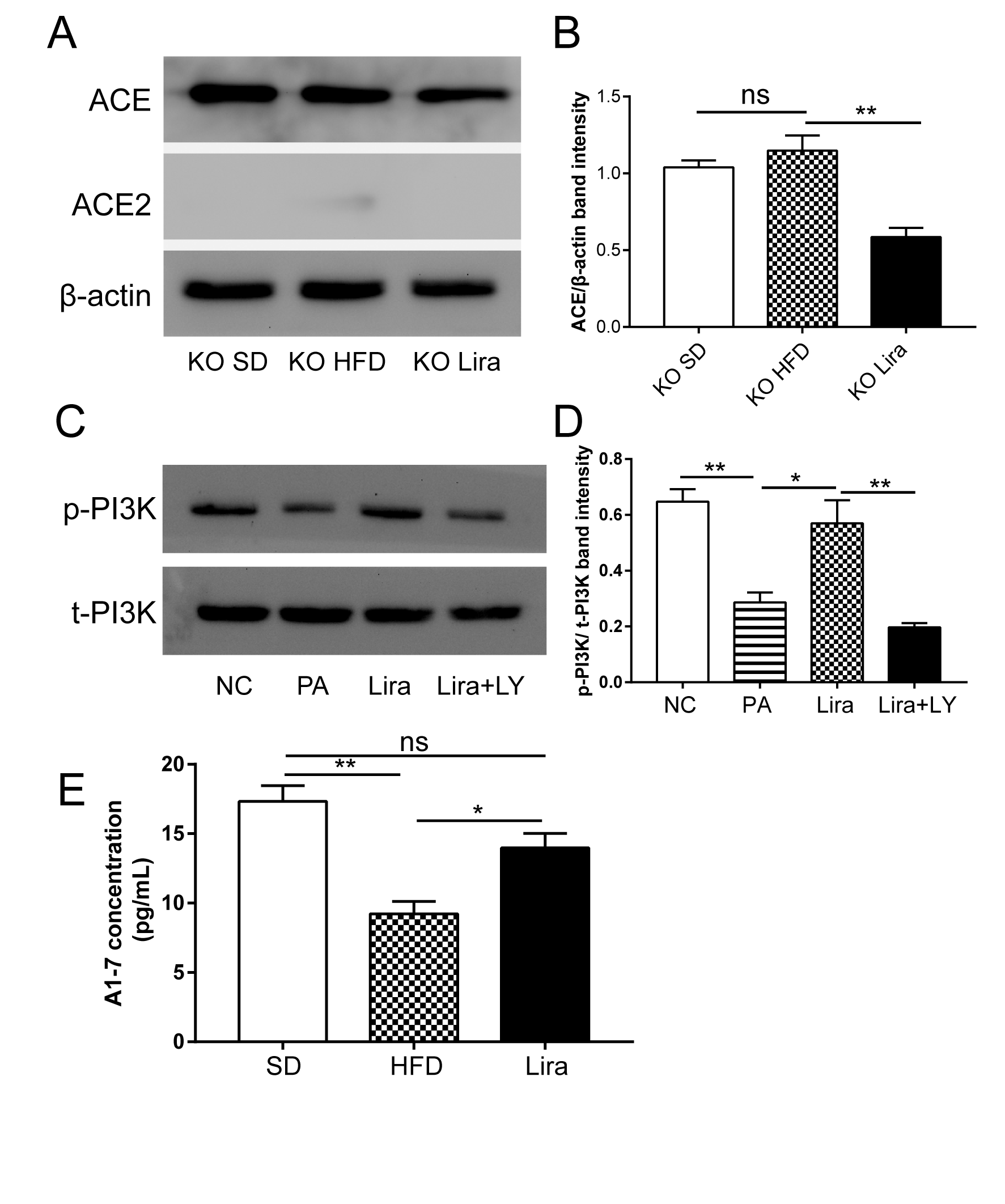

Supplement: Supplementary figure — (A, B) Representative ACE and ACE2 Western blotting images (A) and analysis of ACE protein in livers of ACE2KO mice treated with a HFD or/and liraglutide (B). (C, D) Representative Western blotting images of PI3K phosphorylation protein in HepG2 cells (C) and statistical analysis (D). (E) The plasma concentration of Ang1-7 of WT mice treated with a HFD or/and liraglutide. Data are expressed as the mean ± SD. Ns, no statistical difference; *P < 0.05 and **P < 0.01. [file Image_1.tif]
